# Function-specific repetitive transcranial magnetic stimulation for fine motor rehabilitation in chronic stroke: a case report

**DOI:** 10.1093/psyrad/kkaf033

**Published:** 2025-11-18

**Authors:** Jia-Jia Qi, Hong Li, Kang-Jia Chen, Bing-Bing Zhou, Zu-Juan Ye, Qian Zhou, Jia-Hui Liu, Zhi-Yang Liu, Jue Wang

**Affiliations:** Institute of Sports Medicine and Health, Chengdu Sport University, Chengdu 641418, China; Institute of Sports Medicine and Health, Chengdu Sport University, Chengdu 641418, China; School of Life Science and Technology, University of Electronic Science and Technology of China, Chengdu 611731, China; Institute of Sports Medicine and Health, Chengdu Sport University, Chengdu 641418, China; Institute of Sports Medicine and Health, Chengdu Sport University, Chengdu 641418, China; Institute of Sports Medicine and Health, Chengdu Sport University, Chengdu 641418, China; Institute of Sports Medicine and Health, Chengdu Sport University, Chengdu 641418, China; Institute of Sports Medicine and Health, Chengdu Sport University, Chengdu 641418, China; Institute of Sports Medicine and Health, Chengdu Sport University, Chengdu 641418, China

**Keywords:** fMRI, function-specific rTMS, stroke, fine motor control, hand function, rehabilitation

## Abstract

Although repetitive transcranial magnetic stimulation (rTMS) has been widely used in the treatment of post-stroke hemiparesis, its efficacy in restoring fine motor function of the hand remains limited, largely because of a lack of specificity in target selection. This study presents the case of a 61-year-old female chronic stroke patient who showed significant improvement in hand fine motor function following an 8-week intervention using function-specific rTMS guided by functional magnetic resonance imaging (fMRI). The patient previously experienced multiple lacunar infarctions in the bilateral basal ganglia and centrum semiovale and received the intervention 4 years after the stroke. Following treatment, substantial improvements were observed in fine motor function, with electrophysiological and MRI findings indicating a trend toward restored interhemispheric functional balance. This case highlights a novel therapeutic approach for patients who have shown limited responses to conventional rehabilitation.

## Introduction

Stroke is a cerebrovascular disease that often results in a degree of persistent motor dysfunction, with over 50% of patients continuing to experience hand function impairment 3–6 months after onset (Abo *et al*., 2014; Arwert *et al*., 2018). Currently, physical therapy (Gündüz and Toprak, 2019), occupational therapy (Repšaitė *et al*., 2015), and electrical stimulation (Moon *et al*., 2021) are widely applied methods for the rehabilitation of hand and upper limb motor functions following stroke. These approaches have been shown to be effective for enhancing muscle strength, motor control, and fine motor skills, particularly in the early stages of recovery, when the nervous system exhibits the highest potential for motor function reconstruction. However, the effectiveness of these conventional rehabilitation methods varies considerably because of differences in stroke type, lesion location, and patient-specific characteristics. Consequently, their benefits typically manifest within the first year following stroke onset. Moreover, these interventions are generally more effective in restoring gross motor functions, whereas improvements in fine motor skills of the hand remain relatively limited (Repšaitė *et al*., 2015; Moon *et al*., 2021). Indeed, the proportion of stroke patients achieving complete recovery of fine motor function in the hand is low, estimated at only 5–10% (Gündüz and Toprak, 2019).

Repetitive transcranial magnetic stimulation (rTMS) has been applied to address post-stroke motor dysfunction through modulation of cortical excitability (Hallett, 2007; Feng *et al*., 2023), although its therapeutic effectiveness remains under debate (Tosun *et al*., 2017). Previous studies typically utilized the “hand motor hotspot” as the stimulation target for rTMS interventions. For instance, a single-case study involved a patient who experienced persistent upper limb motor impairment 1 year post-stroke. Researchers applied bilateral stimulation targeting the hand motor hotspots, delivering 1-Hz low-frequency rTMS to the unaffected hemisphere during the initial 7 days, followed by 10-Hz high-frequency stimulation to the affected hemisphere over the subsequent 7 days. This 14-day intervention was integrated with conventional rehabilitation methods, including strength training, range-of-motion exercises, and acupuncture. Although the patient demonstrated an overall improvement in upper limb motor function, improvements in fine motor control of the hand remained limited (Chen *et al*., 2023). Moreover, evidence from cohort studies also indicates that the therapeutic efficacy of rTMS targeting the hand motor hotspot remains inconsistent (Long *et al*., 2018; Abdelkader *et al*., 2024; Xie *et al*., 2025). These findings highlight a critical limitation of targeting strategies based solely on hand motor hotspots: their reliance on eliciting motor-evoked potentials (MEPs) from the affected hemisphere. Consequently, this approach is often challenging to implement in patients with severe motor impairment. To address this limitation, some studies have employed an alternative strategy, using the hand motor hotspot of the unaffected hemisphere and mirroring its corresponding location on the affected hemisphere as the stimulation target. However, the neuroscientific rationale underlying this mirrored targeting approach remains uncertain and lacks robust empirical support. Moreover, these passively evoked “hotspots” do not align with the goal of rehabilitating voluntary motor control (Wang *et al*., 2020).

In contrast, activation points identified through functional magnetic resonance imaging (fMRI) of patients performing finger tapping tasks demonstrate more intensive functional connectivity (FC) with motor cognition-related brain regions than activation points identified with the conventional hotspot approach (Wang *et al*., 2020), and may yield superior regulatory outcomes. FC reflects the neural connectivity and the strength of interactions between brain regions (Hampson *et al*., 2002) and can be used to define function-specific targets by utilizing interhemispheric neural connections, which serve to compensate for damage to corticospinal pathways and modulate the motor area of the affected hemisphere.

## Methods

### Participant

This study was approved by the Ethics Committee of Chengdu Sport University and registered in the Chinese Clinical Trial Registry (ChiCTR2300075705). Written informed consent was obtained from the participant and her son prior to the experiment.

A 61-year-old woman, was admitted to the hospital in April 2020 following sudden-onset right-sided limb weakness and slurred speech. Initial head computed tomography (CT) scans revealed multiple lacunar infarctions in both basal ganglia and the centrum semiovale. CT angiography (CTA) further identified an occlusion in the M1 segment of the left middle cerebral artery. The patient had a history of hypertension. Intravenous thrombolysis with alteplase was ineffective, necessitating a subsequent stent-retriever thrombectomy. Postoperative management included dual antiplatelet therapy (aspirin and clopidogrel) combined with probucol and rosuvastatin to lower lipid levels and stabilize plaque. Additional pharmacological interventions included edaravone for free radical scavenging, Xueshuantong for improving microcirculation, and Xingnaojing to enhance neurological arousal. The patient also received antihypertensive medications, hepatoprotective treatment, and enteral nutritional support. Upon admission, the patient’s National Institutes of Health Stroke Scale (NIHSS) score was 15. By discharge, her clinical condition showed notable improvement, with the NIHSS score having decreased to 5. Although she remained alert, residual neurological deficits persisted, including partial mixed aphasia, facial asymmetry with a flattened right nasolabial fold, deviation of the mouth toward the left, and tongue protrusion deviating to the right. Additionally, voluntary movements of the right limbs were absent and muscle strength was reduced (graded as 2–3 in the right upper limb and 3+ in the right lower limb), while muscle tone in the left limbs remained normal. Following hospital discharge, the patient continued rehabilitation training alongside pharmacological treatments, including antiplatelet agents, antihypertensive and lipid-lowering medications, nutritional support, and hepatoprotective therapy. Detailed clinical information is provided in the Supplementary File (Table S5).

Although there was some early recovery, significant motor impairment persisted on the right side, particularly fine motor function of the hand. By May 2024, when the patient enrolled in this study, conventional rehabilitation had yielded no substantial improvement.

### Study design

Function-specific rTMS targets were defined using both task-based fMRI (Task-fMRI) and resting-state fMRI (RS-fMRI). The target in the unaffected hemisphere was determined based on the activation peak during a finger tapping task, while the target in the affected hemisphere was identified as the peak voxel of activation-based FC within the motor region.

The rTMS intervention lasted for 8 weeks (40 sessions), with one session per day, five days per week, and bilateral stimulation applied. Low-frequency rTMS (1 Hz, 1100 pulses per session) was delivered to the target in the unaffected hemisphere, while high-frequency rTMS (10 Hz, 1000 pulses per session) was applied to the target in the affected hemisphere. Conventional hand rehabilitation training was administered concurrently.

Electrophysiological parameters including resting motor threshold (RMT), central motor conduction time (CMCT), cortical silent period (CSP), and long-interval intracortical inhibition (LICI) were measured daily throughout the intervention. To avoid cumulative effects, the inter-trial interval between each single-pulse stimulation was maintained at at least 1 min (Rossini *et al*., 2015). All procedures were conducted in strict accordance with standardized protocols to ensure data quality. RS-fMRI and Task-fMRI scans were conducted at three time points: pre-intervention (5 May 2024), at 4 weeks (31 May 2024), and post-intervention at 8 weeks (29 June 2024). Upper limb motor function was assessed at each time point using multiple metrics, including joint range of motion, spasticity, muscle strength, grip and pinch strength of the affected hand, the Wolf Motor Function Test (WMFT), and the Fugl–Meyer Assessment of Upper Extremity (FMA-UE).

### MRI acquisition

MRI data were collected at the Qingshuihe Campus of the University of Electronic Science and Technology of China using a 3T GE MR750 scanner. A 3D T1-weighted structural image was acquired using a spoiled gradient recalled echo (SPGR) sequence, with the following parameters: sagittal slices, repetition time (TR) = 8.2 ms, echo time (TE) = 2.98 ms, flip angle (FA) = 8°, matrix size = 256 × 256, field of view (FOV) = 256 × 256 mm, slice thickness = 1 mm with no inter-slice gap, voxel size = 1 × 1 × 1 mm, and 166 slices covering the whole brain. RS- and Task-fMRI images were acquired using a gradient-echo echo-planar imaging (GRE-EPI) sequence. The scanning parameters were: TR = 2000 ms, TE = 30 ms, FA = 90°, 41 contiguous axial slices with no inter-slice gap, matrix size = 64 × 64, FOV = 220 × 220 mm, and voxel size = 3.44 × 3.44 × 3.2 mm. The fMRI scanning protocol included three acquisitions in the following order: a 3D structural scan, an 8.5-min RS-fMRI scan, and a 4-min Task-fMRI scan. A towel was placed between the participant’s head and the coil to enhance comfort and minimize head movement.

During the RS-fMRI scan, the participant was instructed to lie supine on the scanner bed, keep her eyes closed, relax, remain as still as possible, and avoid falling asleep. During the Task-fMRI scan, the participant performed a 4-min block-design task consisting of 30-s blocks. When an instruction image appeared on the screen, the participant was instructed to perform a thumb-to-index finger pinch with the unaffected hand at a rate of once every 2 s. When a fixation cross was displayed, the participant was asked to relax and focus on the center of the screen.

### Data analysis (target definition)

The target in the unaffected hemisphere was identified as the activation peak from the finger tapping task in the first Task-fMRI session. The Task-fMRI data were preprocessed using SPM12 (http://www.fil.ion.ucl.ac.uk/spm/software/spm12/), and included the following steps: slice-timing correction and motion correction, co-registration of functional and structural images, spatial smoothing with a 6-mm full width at half maximum (FWHM) Gaussian kernel, and first-level linear modeling to generate an activation map. A precentral gyrus mask (the site of the primary motor cortex), extracted from the Harvard–Oxford atlas in FSL (http://www.fmrib.ox.ac.uk/fsl), was used to constrain the location of the participant’s activation peak. This mask was co-registered to the participant’s structural image, and the peak activation voxel within the mask was identified as the function-specific target for the unaffected hemisphere. Targets for the 1–4-week and 5–8-week periods were determined based on the first and second Task-fMRI sessions, respectively. Additionally, family-wise error (FWE) correction was applied with *P* < 0.05.

The target in the affected hemisphere was defined as the voxel showing peak FC between the unaffected hemisphere’s target and the lateral motor cortex in the affected hemisphere. FC was calculated using the Data Processing Assistant for Resting-State fMRI (DPARSF) toolbox within the Data Processing & Analysis for Brain Imaging (DPABI) software package (Yan *et al*., 2016), with the peak voxel of task activation from the unaffected hemisphere serving as the seed. A region of interest with a 1-mm radius was centered on the seed to compute whole-brain FC. The peak voxel within the precentral gyrus mask on the affected side that exhibited the strongest FC with the seed was identified as the target in the affected hemisphere. Targets in the affected hemisphere for the 1–4-week and 5–8-week periods were determined based on the corresponding unaffected-side targets from the first and second Task-fMRI sessions, respectively.

### rTMS intervention

To determine the stimulation intensity, the participant’s RMT was measured. The participant sat comfortably in a chair with both hands relaxed and resting on her lap. Surface electromyography was used to record MEPs from the left first dorsal interosseous muscle. Single-pulse stimulation was delivered using a Magstim Super Rapid^2^ (The MAGSTIM Company Ltd, Whitland, UK) stimulator and a 70-mm figure-of-eight coil. RMT was defined as the minimum stimulation intensity required to elicit MEPs with an amplitude of at least 50 μV in five out of 10 consecutive trials. Stimulation intensity was then set to 100% of the individual’s RMT. The BrainSight TMS navigation system (Rogue Research, Montreal, Canada) was used to localize the rTMS target. During stimulation, the neuronavigation system continuously monitored the position of the coil to ensure the stimulation focus was accurately maintained over the target. The coil was positioned at 45° angle from the interhemispheric midline, with the handle oriented backward (Lefaucheur, 2019). All TMS procedures were performed following standardized protocols under the guidance of the neuronavigation system, with the targeting error consistently maintained within 1 mm to ensure precise stimulation localization. To account for dynamic physiological changes in the participant, the RMT was reassessed prior to each treatment session.

### Data analysis (evaluation of brain activity regulation outcomes)

Electrophysiological data analysis: data points exceeding ±2SD were excluded to remove extreme outliers. The locally weighted scatterplot smoothing (LOWESS) method was employed to analyze trends in electrophysiological parameters, allowing for visualization of their dynamic changes throughout the intervention period. The temporal changes of each electrophysiological parameter were assessed using linear regression analysis, with time (days of intervention) as the independent variable and the electrophysiological parameter as the dependent variable. The significance of the regression slope was tested against the null hypothesis of a slope equal to zero to evaluate the statistical significance of parameter changes over the intervention period.

MRI data analysis: RS-fMRI data were preprocessed using SPM12 (http://www.fil.ion.ucl.ac.uk/spm/software/spm12/). The preprocessing steps included: (i) discarding the first 10 time points; (ii) correcting for slice timing differences; (iii) correcting for head motion; (iv) removing nuisance signals (including those from white matter, cerebrospinal fluid, rigid-body six motion parameters, and linear trends); (v) normalizing the functional images to Montreal Neurological Institute (MNI) space using the EPI template in SPM12; (vi) applying spatial smoothing using a Gaussian kernel with an FWHM of 6 mm; and (vii) performing band-pass filtering (0.01–0.1 Hz). Following preprocessing, regional homogeneity (ReHo) and amplitude of low-frequency fluctuation (ALFF) were calculated using the DPARSF toolbox within the DPABI software package (Yan *et al*., 2016).

To assess interhemispheric connectivity, FC was calculated between bilateral M1 regions of interest (ROIs), each including the function-specific target and its mirrored point in the opposite hemisphere. FC between these two ROIs was calculated using the Pearson correlation coefficient of their time series using the DPABI software package (Yan *et al*., 2016).

Clinical indicators: changes in clinical indicators were assessed before and after the intervention.

## Results

All stimulations were well tolerated throughout the intervention period, and no adverse events occurred. After 8 weeks of treatment, the patient exhibited substantial improvements in motor function, including increased joint flexibility, reduced spasticity, and enhanced strength in the affected hand. The FMA-UE score improved from 36 to 51, demonstrating progress in movements both within and outside typical muscle synergy patterns. Specifically, tasks previously unachievable, such as positioning the shoulder at 0° with the elbow at 90° while performing forearm pronation and supination; shoulder abduction at 90° with the elbow extended and forearm pronation; overhead shoulder flexion with elbow extended combined with forearm pronation and supination; and shoulder flexion between 30° and 90° with elbow extended and accompanied by forearm pronation and supination, became partially achievable, with task scores improving from 0 to 1. Additionally, marked improvements were observed in wrist stability and control. Tasks involving wrist dorsiflexion and palmar flexion with the shoulder at 0° and elbow at 90°, and wrist flexion and extension with the shoulder at 30° with elbow extended, yielded higher scores post-intervention, reflecting enhanced wrist functionality. Hand function also improved substantially. Performance scores for tasks such as group extension, hook grasp, pinch grasp, cylindrical grasp, and spherical grasp increased from 0 to 1 or 2, indicating a meaningful recovery of fine motor control. Furthermore, coordination and speed improved, as demonstrated by an increase in the speed score from 1 to 2. The WMFT score increased from 24 to 37, with observable improvements in 10 of the 17 assessed tasks. Specifically, enhanced performance was noted in movements such as forearm-to-table, forearm-to-box, hand-to-table, hand-to-box, and lifting a 1.35-kg basket. Prior to treatment, tasks such as elbow extension, elbow extension with a 0.45-kg object, and elbow flexion to retrieve an object were unachievable. Following intervention, the patient successfully completed these tasks, albeit slowly and with some difficulty. Additionally, performance in the “turn the key in lock” task improved, with the score increasing from 1 to 2, indicating greater hand strength and functional capacity. Figures illustrating the FMA-UE and WMFT, and analyses of percentage change, effect size, and minimal clinically important difference (MCID), are available in the Supplementary File (Figures S1 and S2 and Table S6).

**Figure 1:**
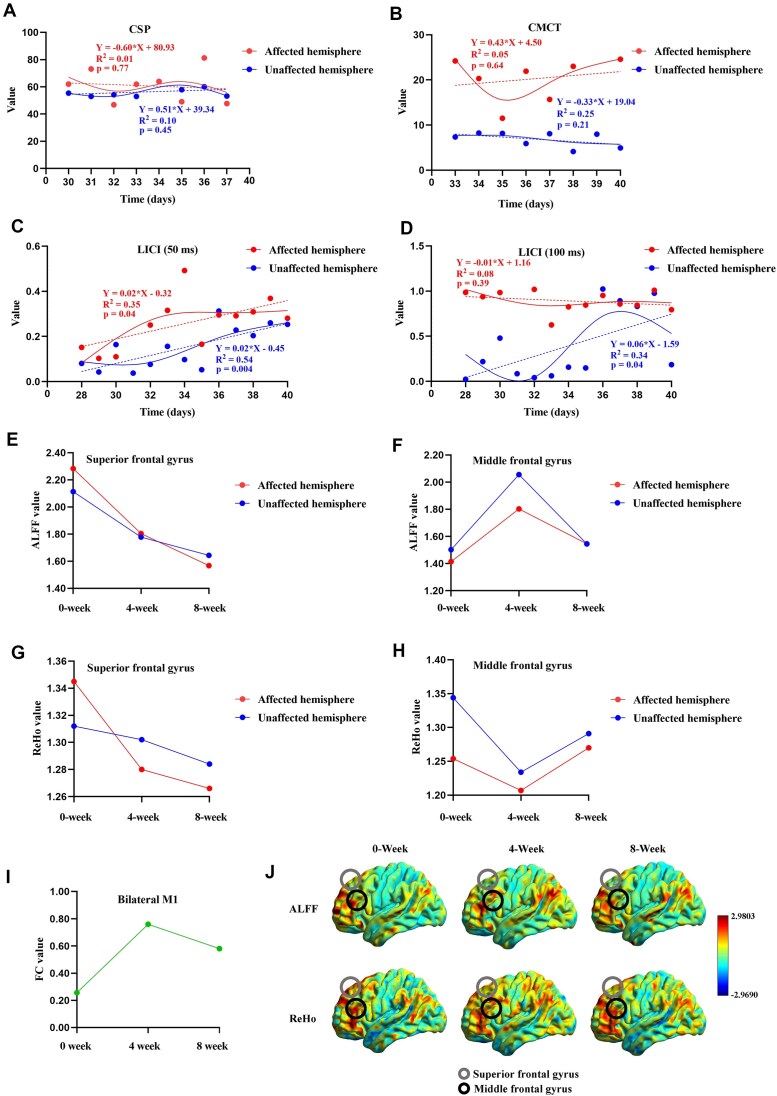
(**A**) Trends in CSP changes in the affected and unaffected hemispheres. (**B**) Trends in CMCT changes in the affected and unaffected hemispheres. (**C**) Trends in LICI (50 ms) changes in the affected and unaffected hemispheres. (**D**) Trends in LICI (100 ms) changes in the affected and unaffected hemispheres. A–D display fitted linear regression lines, with the coefficients of determination (*R*²) and corresponding regression *P*-values indicated on each panel. (**E**) ALFF values in the affected and unaffected superior frontal gyrus at weeks 0, 4, and 8. (**F**) ALFF values in the affected and unaffected middle frontal gyrus at weeks 0, 4, and 8. (**G**) ReHo values in the affected and unaffected superior frontal gyrus at weeks 0, 4, and 8. (**H**) ReHo values in the affected and unaffected middle frontal gyrus at weeks 0, 4, and 8. (**I**) FC values in bilateral M1 at weeks 0, 4, and 8 (voxel‐level *P* < 0.001, uncorrected). (**J**) Whole-brain distributions of ALFF and ReHo at baseline (week 0), week 4, and week 8. CSP, cortical silent period; CMCT, central motor conduction time; LICI, long-interval intracortical inhibition; ALFF, amplitude of low-frequency fluctuation; ReHo, regional homogeneity; FC, functional connectivity.

Electrophysiological data revealed a shortened CSP on the affected side (Fig. 1A); the CMCT initially decreased and then slightly increased (Fig. 1B). LICI (50 ms) approached the level of the unaffected side, while LICI (100 ms) fluctuated around 1 (Fig. 1C–D). The linear regression results showed that the slopes were significant for LICI (50 ms) in both the affected and unaffected hemispheres, as well as for LICI (100 ms) in the unaffected hemisphere (*P* < 0.05; Fig. 1C–D), whereas the slopes for CSP and CMCT in both hemispheres, as well as for LICI (100 ms) in the affected hemisphere, were not significant (*P* > 0.05; Fig. 1A–B, D). Daily data on electrophysiological parameters during the intervention are provided in the Supplementary File (Tables S7 and S8).

**Figure 2:**
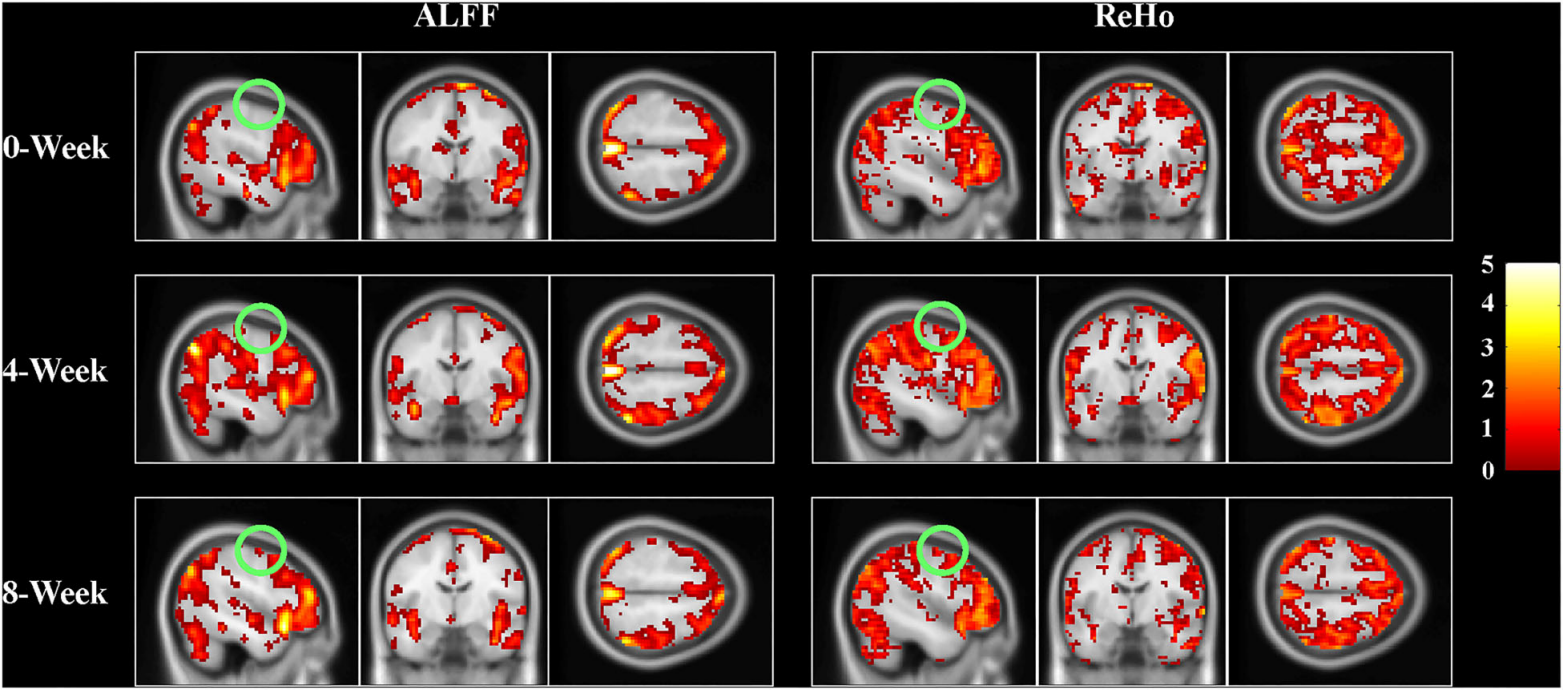
ALFF and ReHo maps with FDR correction for multiple comparisons (*P* < 0.05, cluster size ≥ 10 voxels) at baseline (week 0), week 4, and week 8. ALFF, amplitude of low-frequency fluctuation; ReHo, regional homogeneity; FDR, false discovery rate.

fMRI results demonstrated improved functional activity in the affected hemisphere, particularly in the frontal gyrus, where both ALFF and ReHo became more similar to those of the unaffected side (Fig. 1E–H). Whole-brain trends in ALFF and ReHo are presented in Fig. 1J. ALFF and ReHo showed an initial increase followed by a decrease at weeks 0, 4, and 8 (Fig. 2). However, the overall levels remained higher than the baseline (week 0). FC between the bilateral M1 ROIs was 0.26 at baseline, increased to 0.76 at week 4, and decreased to 0.58 at week 8. (Fig. 1I). These findings suggest that function-related brain activity was activated during the early phase of the intervention and gradually stabilized over time (Figs 1I and 2). Detailed clinical outcome measures and a video illustrating the rehabilitation effects are provided in the Supplementary File.

## Discussion

The findings of this study support the efficacy of function-specific rTMS for improving hand function in patients with chronic stroke, particularly among those who have shown limited responsiveness to conventional rehabilitation. Under normal conditions, the corpus callosum connects the two cerebral hemispheres and facilitates a dynamic balance between excitatory and inhibitory neural activity. However, this interhemispheric balance may be disrupted following stroke; according to the interhemispheric competition model (Boddington and Reynolds, 2017), stroke-induced damage leads to a marked reduction in excitability within the affected hemisphere. This in turn weakens transcallosal inhibition of the unaffected hemisphere, resulting in hyperexcitability on the unaffected side. This excessive activity of the unaffected hemisphere further inhibits the already compromised affected hemisphere, creating a self-reinforcing cycle of interhemispheric imbalance. rTMS can help restore this balance through frequency-specific modulation. Low-frequency rTMS applied to the unaffected hemisphere can reduce its excitability and mitigate its excessive inhibitory influence. Conversely, high-frequency rTMS applied to the affected hemisphere can enhance its excitability. Together, these interventions may help re-establish a more normalized pattern of interhemispheric interaction, thereby supporting functional recovery.

Our electrophysiological data demonstrate that the LICI (50 ms) values in the affected hemisphere approached those of the unaffected side, accompanied by a reduction in CSP duration, suggesting restored cortical excitability with diminished inhibition and enhanced facilitation, reflecting a gradual restoration of interhemispheric balance.

The fMRI results revealed that ALFF and ReHo values in the frontal lobe of the affected hemisphere became closer to those of the unaffected side following treatment, suggesting functional recovery in motor-related brain regions. The frontal lobe plays a critical role in motor planning, fine motor control, and attentional regulation (Briggs *et al*., 2020, 2021), and its functional reorganization may contribute to improved motor outcomes. ALFF, ReHo and FC levels were higher at week 4 of the intervention compared with both baseline and week 8, followed by a relatively stable state at week 8 (Figs 1I and 2). This temporal trajectory suggests an early phase of heightened neural activity, transitioning into a stage characterized by functional integration and optimization. Such a pattern aligns with the “cortical maturation” theory observed in brain development. According to this theory, the early expansion of white matter is associated with increased neuronal activity and synaptic connectivity, facilitating widespread functional reorganization. In contrast, the subsequent phase of cortical thinning reflects synaptic pruning and metabolic efficiency, ultimately supporting more refined and efficient functional regulation (Sowell *et al*., 2001, 2003, 2004; Gogtay *et al*., 2004; Wang *et al*., 2015). Based on this framework, we propose that the observed increase in ALFF, ReHo, and FC, followed by stabilization, may represent dynamic regulation of interhemispheric brain function mediated by transcallosal connectivity. Under rTMS intervention, the brain may initially exhibit a phase of elevated functional activation, which is then followed by progressive integration and stabilization, ultimately contributing to functional recovery.

The majority of studies investigating rTMS for post-stroke motor function recovery have employed unilateral stimulation protocols, and research exploring bilateral rTMS interventions remains relatively scarce. A 2025 meta-analysis by Xie et al. encompassing 70 studies revealed that only two utilized bilateral rTMS protocols. Of the remainder, 54 adopted the conventional hand motor hotspot as the stimulation target, while three determined targets based on anatomical landmarks on the scalp (Xie *et al*., 2025).

Long *et al*. (2018) found that bilateral rTMS targeting the hand motor hotspot in the unaffected hemisphere and its mirrored location in the affected hemisphere more effectively promoted upper limb motor recovery than did unilateral stimulation. However, a randomized controlled trial by Abdelkader *et al*. (2024) compared bilateral and unilateral rTMS, both targeting the hand motor hotspot, and reported no significant difference in motor function improvement between the two approaches. These inconsistent modulatory effects suggest that treatment outcomes may be influenced not only by the laterality of stimulation, but also by the specificity of target selection.

Despite increasing interest in individualized neuromodulation, studies using task-based fMRI to guide rTMS target localization remain rare. We have identified only one such study published between 2023 and 2025, which focused on post-stroke dysphagia (Chen *et al*., 2024). Guided by the interhemispheric competition model, we here applied bilateral rTMS and introduced a new method based on function-specific targeting.

In this study, we combined Task-fMRI and resting-state FC analysis to define individualized function-specific targets in both cerebral hemispheres. In the unaffected hemisphere, the target was identified as the activation peak elicited during a finger tapping task. In the affected hemisphere, the corresponding target was defined as the voxel showing the strongest FC with that peak in the unaffected hemisphere. Our previous research (Wang *et al*., 2020) demonstrated that such fMRI-guided targets are typically located more laterally than the conventional hand motor hotspot and exhibit more intensive FC with motor-cognition-related regions, including the premotor cortex, putamen, insula, and globus pallidus. These findings suggest that fMRI-guided targets may be more appropriate for modulation of voluntary motor control.

During the intervention, both the participant’s brain activation patterns and affected-side FC changed, accompanied by gradual improvements in hand function. Following the principle of function-specific targeting, stimulation sites were adjusted at different stages to ensure that rTMS consistently focused on the most relevant hand motor regions. Specifically, each fMRI session involved a finger tapping task, and targets were determined according to the peak voxel of task activation, which differed between sessions: the unaffected-side target shifted from (42, 18, 29) to (45, 18, 40), and the affected-side target shifted from (–51, 36, 10) to (–59, 22, 17). These stage-specific adjustments not only considered changes in clinical function but also reflected functional alterations in post-stroke motor cortical state.

Individualized, function-specific rTMS represents a promising therapeutic strategy for chronic stroke patients. However, larger-scale studies are necessary to validate its generalizability. Furthermore, the approach holds potential for broader application in other neurological disorders, advancing the field of neurorehabilitation.

## Limitations

Although this study provides preliminary evidence supporting the efficacy of fMRI-based, function-specific, individualized rTMS intervention in stroke patients, its generalizability remains to be established because this study is based on a single case. The inherent constraints of a single-subject study prevent a comprehensive understanding of interindividual variability in response to an intervention. Moreover, the single-subject design does not allow direct inference of the test–retest reliability of ALFF, ReHo, and FC from the current dataset. Additionally, formal long-term follow-up assessments were not conducted. Future studies should include larger cohorts of stroke patients, extend the duration of rTMS intervention, and implement systematic long-term follow-up to better evaluate the broader applicability of this approach and facilitate the integration of rTMS into clinical practice.

## Supplementary Material

kkaf033_Supplemental_Files

## References

[bib1] Abdelkader AA, Afifi LM, Maher EA et al. (2024) Comparison of bilateral versus unilateral 5 hz or 1 hz repetitive transcranial magnetic stimulation in subacute stroke: assessment of motor function in a randomized controlled study. J Clin Neurophysiol. 41:478–83. 38935659 10.1097/WNP.0000000000000987

[bib2] Abo M, Kakuda W, Momosaki R et al. (2014) Randomized, multicenter, comparative study of NEURO versus CIMT in poststroke patients with upper limb hemiparesis: the NEURO-VERIFY Study. Int J Stroke. 9:607–12. 24015934 10.1111/ijs.12100

[bib3] Arwert H, Schut S, Boiten J et al. (2018) Patient reported outcomes of hand function three years after stroke. Top Stroke Rehabil. 25:13–9. 29025365 10.1080/10749357.2017.1385232

[bib4] Boddington LJ, Reynolds JNJ (2017) Targeting interhemispheric inhibition with neuromodulation to enhance stroke rehabilitation. Brain Stimul. 10:214–22. 28117178 10.1016/j.brs.2017.01.006

[bib5] Briggs RG, Khan AB, Chakraborty AR et al. (2020) Anatomy and white matter connections of the superior frontal gyrus. Clin Anat. 33:823–32. 31749198 10.1002/ca.23523

[bib6] Briggs RG, Lin Y-H, Dadario NB et al. (2021) Anatomy and white matter connections of the middle frontal gyrus. World Neurosurg. 150:e520–9. 33744423 10.1016/j.wneu.2021.03.045

[bib7] Chen M, Huang Z, Chen Y et al. (2024) Repetitive transcranial magnetic stimulation on individualized spots based on task functional magnetic resonance imaging improves swallowing function in poststroke dysphagia. Brain Connect. 14:513–26. 39302050 10.1089/brain.2024.0021

[bib8] Chen N, Qiu X, Hua Y et al. (2023) Effects of sequential inhibitory and facilitatory repetitive transcranial magnetic stimulation on neurological and functional recovery of a patient with chronic stroke: a case report and literature review. Front Neurol. 14:1064718. 36779047 10.3389/fneur.2023.1064718 PMC9911674

[bib9] Feng W, Plow EB, Paik NJ (2023) Transcranial magnetic stimulation for poststroke motor recovery: what we have learned. Stroke. 54:1972–3. 37345547 10.1161/STROKEAHA.123.043536

[bib10] Gogtay N, Giedd JN, Lusk L et al. (2004) Dynamic mapping of human cortical development during childhood through early adulthood. Proc Natl Acad Sci USA. 101:8174–9. 15148381 10.1073/pnas.0402680101 PMC419576

[bib11] Gündüz O.H., Toprak, C.​​​​​​ Şanal. (2019). Hand Function in Stroke. In: Duruöz M. T. (ed). Hand Function: A Practical Guide to Assessment, 2nd edn. Cham: Springer, 125–135.

[bib12] Hallett M (2007) Transcranial magnetic stimulation: a primer. Neuron. 55:187–99. 17640522 10.1016/j.neuron.2007.06.026

[bib13] Hampson M, Peterson BS, Skudlarski P et al. (2002) Detection of functional connectivity using temporal correlations in MR images. Hum Brain Mapp. 15:247–62. 11835612 10.1002/hbm.10022 PMC6872035

[bib14] Lefaucheur JP (2019) Transcranial magnetic stimulation. Handb Clin Neurol. 160:559–80. 31277876 10.1016/B978-0-444-64032-1.00037-0

[bib15] Long H, Wang H, Zhao C et al. (2018) Effects of combining high- and low-frequency repetitive transcranial magnetic stimulation on upper limb hemiparesis in the early phase of stroke. Restor Neurol Neurosci. 36:21–30. 29439359 10.3233/RNN-170733

[bib16] Moon JH, Cho HY, Hahm SC (2021) Influence of electrotherapy with task-oriented training on spasticity, hand function, upper limb function, and activities of daily living in patients with subacute stroke: a double-blinded, randomized, controlled trial. Healthcare (Basel). 9. 987 34442124 10.3390/healthcare9080987 PMC8392129

[bib17] Repšaitė V, Vainoras A, Berškienė K et al. (2015) The effect of differential training-based occupational therapy on hand and arm function in patients after stroke: results of the pilot study. Neurol Neurochir Pol. 49:150–5. 26048602 10.1016/j.pjnns.2015.04.001

[bib18] Rossini PM, Burke D, Chen R et al. (2015) Non-invasive electrical and magnetic stimulation of the brain, spinal cord, roots and peripheral nerves: basic principles and procedures for routine clinical and research application. An updated report from an I.F.C.N. Committee. Clin Neurophysiol. 126:1071–107. 25797650 10.1016/j.clinph.2015.02.001 PMC6350257

[bib19] Sowell ER, Peterson BS, Thompson PM et al. (2003) Mapping cortical change across the human life span. Nat Neurosci. 6:309–15. 12548289 10.1038/nn1008

[bib20] Sowell ER, Thompson PM, Leonard CM et al. (2004) Longitudinal mapping of cortical thickness and brain growth in normal children. J Neurosci. 24:8223–31. 15385605 10.1523/JNEUROSCI.1798-04.2004 PMC6729679

[bib21] Sowell ER, Thompson PM, Tessner KD et al. (2001) Mapping continued brain growth and gray matter density reduction in dorsal frontal cortex: inverse relationships during postadolescent brain maturation. J Neurosci. 21:8819–29. 11698594 10.1523/JNEUROSCI.21-22-08819.2001 PMC6762261

[bib22] Tosun A, Türe S, Askin A et al. (2017) Effects of low-frequency repetitive transcranial magnetic stimulation and neuromuscular electrical stimulation on upper extremity motor recovery in the early period after stroke: a preliminary study. Top Stroke Rehabil. 24:361–7. 28327054 10.1080/10749357.2017.1305644

[bib23] Wang J, Meng H-J, Ji G-J et al. (2020) Finger tapping task activation vs. TMS hotspot: different locations and networks. Brain Topogr. 33:123–34. 31691912 10.1007/s10548-019-00741-9 PMC6943404

[bib24] Wang J, Yang N, Liao W et al. (2015) Dorsal anterior cingulate cortex in typically developing children: laterality analysis. Dev Cogn Neurosci. 15:117–29. 26602957 10.1016/j.dcn.2015.10.002 PMC6989820

[bib25] Xie G, Wang T, Deng L et al. (2025) Repetitive transcranial magnetic stimulation for motor function in stroke: a systematic review and meta-analysis of randomized controlled studies. Syst Rev. 14:47. 39994795 10.1186/s13643-025-02794-3 PMC11849290

[bib26] Yan C-G, Wang X-D, Zuo X-N et al. (2016) DPABI: data processing & analysis for (resting-state) brain imaging. Neuroinformatics. 14:339–51. 27075850 10.1007/s12021-016-9299-4

