# Supplementary material for: Function-specific repetitive transcranial magnetic stimulation for fine motor rehabilitation in chronic stroke: a case report
Source: Psychoradiology. 2025 Nov 18;6:kkaf033. doi: 10.1093/psyrad/kkaf033 (PMC12902691; doi:10.1093/psyrad/kkaf033)
Supplement: kkaf033_Supplemental_Files [file kkaf033_supplemental_files.zip › Supplementary File.docx]

**Function-Specific Repetitive Transcranial Magnetic Stimulation for Fine Motor Rehabilitation in Chronic Stroke: A Case Report**

Jia-Jia Qi^1^#, Hong Li^1^#, Kang-Jia Chen^2^, Bing-Bing Zhou^1^, Zu-Juan Ye^1^, Qian Zhou^1^, Jia-Hui Liu^1^, Zhi-Yang Liu^1^, Jue Wang^1^*

^1^Institute of sports medicine and health, Chengdu Sport University, Chengdu 641418, China

^2^School of Life Science and Technology, University of Electronic Science and Technology of China, Chengdu 611731, China

*Correspondence: Jue Wang, juefirst@cdsu.edu.cn

#These authors contributed equally to this work.

**Table S1.** Range of motion, spasticity, and muscle strength

| **Measurement site** | **Motion** | **Active range of motion (°) (0/4/8-Week)** | **Passive range of motion(°) (0/4/8-Week)** | **Spasticity (0/4/8-Week)** | **Muscle strength (0/4/8-Week)** |
| --- | --- | --- | --- | --- | --- |
| **Shoulder** | Flexion | 70/140/170 | 140/155/180 | 1/1/1 | 4/4/4 |
|  | Extension | 34/50/30 | 70/55/60 | 1+/1+/1+ | 4/4/4 |
|  | Abduction | 80/135/90 | 140/148/140 | 1+/1/1 | 4/4/4 |
|  | Adduction | 60/45/40 | 60/75/65 | 1/1/0 | 4/4/4 |
|  | Internal rotation | 10/10/50 | 75/78/90 | 2/1+/1 | 3/3/4 |
|  | External rotation | 0/10/25 | 70/81/55 | 2/1+/1 | 0/3/4 |
| **Elbow** | Flexion | 135/135/135 | 135/135/145 | 0/0/0 | 4/4/4 |
|  | Extension | 0/0/0 | 0/0/0 | 3/1+/1+ | 1/3/4 |
|  | Internal rotation | 65/65/65 | 85/70/85 | 1/1/0 | 2/2/3 |
|  | External rotation | 15/10/45 | 80/80/95 | 3/1+/1 | 4/3/3 |
| **Wrist** | Radial deviation | 0/0/0 | 60/60/65 | 4/1/0 | 1/1/1 |
|  | Ulnar deviation | 0/0/10 | 20/20/25 | 1/1/0 | 3/3/3 |
|  | Flexion | 5/10/25 | 60/80/60 | 3/1+/0 | 2/2/2 |
|  | Extension | 0/0/0 | 60/65/65 | 1/1+/1+ | 1/1/2 |

**Table S2.** The Fugl-Meyer Assessment of Upper Extremity

| **Functional domain** | **Test item** | **Score (0/8-Week)** |
| --- | --- | --- |
| **Reflex Activity** | Biceps reflex | 2/2 |
|  | Triceps reflex | 2/2 |
| **Flexor Synergy** | Shoulder elevation | 2/2 |
|  | Shoulder retraction | 2/2 |
|  | Shoulder abduction | 2/2 |
|  | Shoulder external rotation | 2/2 |
|  | Elbow flexion | 2/2 |
|  | Forearm supination | 2/2 |
| **Extensor Synergy** | Shoulder adduction and internal rotation | 2/2 |
|  | Elbow extension | 2/2 |
|  | Forearm pronation | 2/2 |
| **Movement within Synergy** | Hand to lumbar spine | 1/2 |
|  | Shoulder flexion 90°, elbow extended | 1/1 |
|  | Shoulder at 0°, elbow at 90°, forearm pronation and supination | 0/1 |
| **Movement out of Synergy** | Shoulder abduction at 90°, elbow extended, forearm pronation | 0/1 |
|  | Shoulder flexion overhead, elbow extended, forearm pronation and supination | 0/1 |
|  | Shoulder flexion 30°–90°, elbow extended, forearm pronation and supination | 0/1 |
| **Hyperreflexia** | Biceps, triceps, and finger flexor reflexes | 1/0 |
| **Wrist Stability** | Shoulder at 0°, elbow at 90°, wrist dorsiflexion | 1/2 |

To be continued

**Table S2.** The Fugl-Meyer Assessment of Upper Extremity

| **Functional domain** | **Test item** | **Score (0/8-Week)** |
| --- | --- | --- |
|  | Shoulder at 0°, elbow at 90°, wrist palmar flexion | 0/2 |
|  | Shoulder at 30°, elbow extended, wrist dorsiflexion | 1/1 |
|  | Shoulder at 30°, elbow extended, wrist flexion and extension | 0/1 |
|  | Shoulder at 30°, elbow extended, wrist circular movement | 0/0 |
| **Hand Function** | Group flexion | 2/2 |
|  | Group extension | 0/1 |
|  | Hook grasp | 0/2 |
|  | Lateral pinch | 2/2 |
|  | Pinch grasp | 0/1 |
|  | Cylindrical grasp | 0/1 |
|  | Spherical grasp | 0/1 |
| **Coordination/Speed** | Tremor | 2/2 |
|  | Disturbance in spatial discrimination | 2/2 |
|  | Speed | 1/2 |
| **Total Score** | | 36/51 |

**Table S3.** The Wolf Motor Function Test

| **Movement Task** | **Score (0/4/8-Week)** |
| --- | --- |
| **Forearm to table** | 3/3/4 |
| **Forearm to box** | 3/3/4 |
| **Extended elbow** | 1/2/3 |
| **Extended elbow with 0.45 kg weight** | 1/2/3 |
| **Hand to table** | 3/3/4 |
| **Hand to box** | 3/3/4 |
| **Hand to box with 0.45 kg weight** | 0.5/0.5/0.5kg |
| **Reach and retrieve 0.45 kg weight by elbow flexion** | 1/3/4 |
| **Lift can to mouth** | 1/1/1 |
| **Lift pencil from table** | 1/1/1 |
| **Lift paper clip from table** | 1/1/1 |
| **Stack 3 checkers** | 1/1/1 |
| **Flip 3 cards** | 1/1/1 |
| **Grip strength** | 6.0/5.4k/6.8kg |
| **Turn the key in lock** | 1/1/2 |
| **Fold towel** | 1/1/1 |
| **Lift basket with 1.35 kg weight** | 2/2/3 |

**Table S4.** Grip strength and pinch strength

| **Strength Type** | **Score (kg) (0/4/8-Week)** |
| --- | --- |
| **Grip strength** | 6.0/5.4/6.8 |
| **Pinch strength** | 6.7/7.0/7.2 |

**Table S5.** Detailed Clinical Information of the Patient

| **Category** | **Item** | **Content** |
| --- | --- | --- |
| **Diagnostic Information** | **Primary Diagnosis** | Acute cerebral infarction with left middle cerebral artery stenosis and multiple lacunar infarctions in the bilateral basal ganglia and centrum semiovale. |
|  | **Comorbidities** | Grade 3 hypertension (very high risk), hypertensive heart disease, abnormal liver function, severe malnutrition, hypokalemia, allergic dermatitis, arterial plaques. |
| **Examination Findings** | **Past Medical History** | Long-standing history of hypertension; admission blood pressure: 220/106 mmHg. |
|  | **Neuroimaging** | MRI and DWI showed multiple acute/subacute infarctions in the left temporo-parieto-occipital lobes and centrum semiovale; possible hemorrhage in the left basal ganglia region; demyelinating changes; MRA showed cerebral arteriosclerosis, multiple focal stenoses, and distal occlusion of the left posterior cerebral artery. |
|  | **Echocardiography** | Left ventricular hypertrophy with normal systolic function and impaired diastolic function. |
|  | **Liver Function** | ALT 141.3 U/L, AST 94.3 U/L. |
|  | **Ambulatory Blood Pressure** | 24-hour average blood pressure of 159/92 mmHg with significant variability. |
| **Inpatient Management** | **Acute Phase Interventions** | Intravenous thrombolysis with alteplase plus stent-retriever thrombectomy and blood pressure control. |
|  | **Antiplatelet Therapy** | Aspirin, clopidogrel. |
|  | **Lipid-lowering/Anti-atherosclerotic Therapy** | Rosuvastatin, probucol. |
|  | **Neuroprotective Therapy** | Edaravone, Xingnaojing injection, Xueshuantong injection. |
|  | **Antihypertensive therapy** | Urapidil, irbesartan, nifedipine. |

To be continued

**Table S5.** Detailed Clinical Information of the Patient Case

| **Category** | **Item** | **Content** |
| --- | --- | --- |
|  | **Supportive Care** | Enteral nutrition, hepatoprotective therapy, anti-allergic therapy, rehabilitation therapy. |
| **Discharge Status** | **Consciousness** | Alert with residual mixed aphasia. |
|  | **Motor Function** | Right upper limb muscle strength grade 2–3, right lower limb grade 3+, normal on the left limbs. |
|  | **Neurological Scores** | NIHSS score 5, mRS score 4. |
|  | **Diet/Activity** | Restricted oral intake and activity; continued rehabilitation needed. |
| **Discharge Medications** | **Antiplatelet Agents** | Aspirin 100 mg nightly, clopidogrel 75 mg nightly (switch to single agent after 10 days). |
|  | **Lipid-lowering Agents** | Rosuvastatin 20 mg nightly, probucol 250 mg twice daily. |
|  | **Antihypertensives** | Irbesartan 150 mg once daily, nifedipine 30 mg once daily. |
|  | **Neurotrophic Agents** | Mecobalamin 0.2 g three times daily. |
|  | **Gastroprotective Agents** | Aluminum-magnesium suspension 15 ml three times daily, rebamipide 0.1 g three times daily. |
|  | **Hepatoprotective Agents** | Continue hepatoprotective therapy, monitor liver function regularly, adjust rosuvastatin as needed. |

MRI, Magnetic Resonance Imaging; DWI, Diffusion Weighted Imaging; MRA, Magnetic Resonance Angiography; ALT, Alanine Aminotransferase; AST, Aspartate Aminotransferase; NIHSS, National Institutes of Health Stroke Scale; mRS, modified Rankin Scale.

The minimal clinically important differences (MCIDs) for the Fugl-Meyer Assessment of Upper Extremity (FMA-UE) and the Wolf Motor Function Test (WMFT) in this study were determined based on the findings of Hiragami et al. 2019(Hiragami et al., 2019) and Lin et al. 2009(Lin et al., 2009).

**Table S6.** Percentage Change, Effect Size, and MCID of FMA-UE Score and Mean WMFT Score After Intervention

| **Assessment** | **Δ Score** | **Percentage Change** | **Effect Size (Cohen’s d)** | **MCID** |
| --- | --- | --- | --- | --- |
| **FMA-UE** | 15 | 41.67% | 1.21 | 12.40 |
| **WMFT** | 0.87 | 54.17% | 2.34 | 0.37 |

MCID, Minimal Clinically Important Difference; FMA-UE, Fugl-Meyer Assessment of Upper Extremity; WMFT, Wolf Motor Function Test.

**Table S7.** RMT, CMCT, CSP, and LICI in the Unaffected Hemisphere During the Intervention

| **Time (days)** | **RMT** | **CMCT** | **CSP** | **LICI (50 ms) (MEP_TS_/MEP_CS_)** | **LICI (100 ms) (MEP_TS_/MEP_CS_)** |
| --- | --- | --- | --- | --- | --- |
| **1** | 68 | 6.90 | 17.91 | 110/840 | 134/969 |
| **2** | 53 | 11.00 | 26.19 | 240/262 | 233/244 |
| **3** | 66 | 3.03 | 51.80 | 90/712 | 52/107 |
| **4** | 60 | 11.41 | 18.92 | 162/185 | 136/206 |
| **5** | 63 | 6.78 | 42.25 | 108/111 | 91/114 |
| **6** | 58 | 12.40 | 33.39 | 222/248 | 208/213 |
| **7** | 60 | 3.43 | 44.69 | 95/111 | 83/84 |
| **8** | 60 | - | - | - | - |
| **9** | 78 | 16.14 | 42.16 | 64/108 | 90/91 |
| **10** | 68 | 7.73 | 43.90 | 224/238 | 254/263 |
| **11** | 68 | 6.70 | 66.29 | 179/404 | 173/340 |
| **12** | 67 | 7.65 | 52.93 | 165/515 | 104/194 |
| **13** | 65 | 9.25 | 54.49 | 39/48 | 37/104 |
| **14** | 60 | 6.26 | 58.81 | 39/531 | 99/707 |
| **15** | 65 | 6.02 | 64.23 | 33/116 | 50/173 |
| **16** | 69 | 8.09 | 60.76 | 81/958 | 98/1320 |
| **17** | 60 | 4.52 | 42.27 | 15/153 | 226/446 |
| **18** | 62 | 8.57 | 64.04 | 220/431 | 251/348 |
| **19** | 60 | 7.15 | 59.57 | 76/505 | 86/93 |
| **20** | 60 | - | - | - | - |
| **21** | 69 | 6.09 | 63.59 | 50/378 | 60/584 |
| **22** | 67 | 6.61 | 56.87 | 15/614 | 6/352 |
| **23** | 67 | 6.47 | 47.36 | 23/709 | 21/519 |
| **24** | 66 | 7.07 | 61.17 | 23/248 | 13/561 |

To be continued

**Table S7.** RMT, CMCT, CSP, and LICI in the Unaffected Hemisphere During the Intervention

| **Time (days)** | **RMT** | **CMCT** | **CSP** | **LICI (50 ms) (MEP_TS_/MEP_CS_)** | **LICI (100 ms) (MEP_TS_/MEP_CS_)** | |
| --- | --- | --- | --- | --- | --- | --- |
| **25** | 74 | 6.64 | 54.86 | 79/381 | 132/244 |  |
| **26** | 65 | 5.33 | 57.20 | 256/263 | 537/610 |  |
| **27** | 65 | 6.00 | 51.58 | 77/417 | 106/672 |  |
| **28** | 67 | 6.62 | 66.82 | 46/569 | 14/567 |  |
| **29** | 70 | 6.79 | 49.22 | 13/303 | 55/251 |  |
| **30** | 66 | 3.94 | 55.36 | 24/146 | 202/421 |  |
| **31** | 71 | 7.36 | 52.95 | 30/793 | 20/234 |  |
| **32** | 68 | 8.35 | 54.16 | 102/1328 | 36/850 |  |
| **33** | 64 | 7.36 | 52.98 | 28/179 | 52/843 |  |
| **34** | 65 | 8.24 | 63.86 | 75/767 | 70/442 |  |
| **35** | 63 | 8.15 | 57.86 | 36/683 | 53/355 |  |
| **36** | 62 | 5.92 | 60.05 | 695/2221 | 843/824 |  |
| **37** | 74 | 8.10 | 53.22 | 87/381 | 75/84 |  |
| **38** | 66 | 4.14 | 57.68 | 21/103 | 64/77 |  |
| **39** | 71 | 7.99 | 46.34 | 71/273 | 124/127 |  |
| **40** | 72 | 4.94 | 45.44 | 67/264 | 91/489 |  |

The “-” indicates that the measurement was not conducted on that day because the participant was undergoing fMRI scanning on another campus.

RMT, Resting motor threshold; CMCT, Central Motor Conduction Time; CSP, Cortical Silent Period; LICI, Long-interval Intracortical Inhibition; MEP, Motor Evoked Potential; TS, Test Stimulus; CS, Conditioning Stimulus.

**Table S8.** CMCT, CSP, and LICI in the Affected Hemisphere During the Intervention

| **Time (days)** | **CMCT** | **CSP** | **LICI (50 ms) (MEP_TS_/MEP_CS_)** | **LICI (100 ms) (MEP_TS_/MEP_CS_)** |
| --- | --- | --- | --- | --- |
| **28** | - | - | 112/738 | 70/71 |
| **29** | - | - | 44/426 | 61/65 |
| **30** | - | 61.99 | 54/490 | 63/64 |
| **31** | - | 73.10 | 54/45 | 72/348 |
| **32** | - | 46.84 | 363/1449 | 106/104 |
| **33** | 24.22 | 61.91 | 36/114 | 15/24 |
| **34** | 20.30 | 63.86 | 32/65 | 33/40 |
| **35** | 11.50 | 49.01 | 24/145 | 22/26 |
| **36** | 21.93 | 81.26 | 62/210 | 20/21 |
| **37** | 15.70 | 47.71 | 28/96 | 24/28 |
| **38** | 23.02 | - | 86/278 | 54/64 |
| **39** | 3.68 | - | 849/2301 | 730/723 |
| **40** | 24.58 | - | 116/413 | 81/102 |

The “-” indicates that the measurement was conducted, but no valid data were obtained.

RMT, Resting motor threshold; CMCT, Central Motor Conduction Time; CSP, Cortical Silent Period; LICI, Long-interval Intracortical Inhibition; MEP, Motor Evoked Potential; TS, Test Stimulus; CS, Conditioning Stimulus.

**
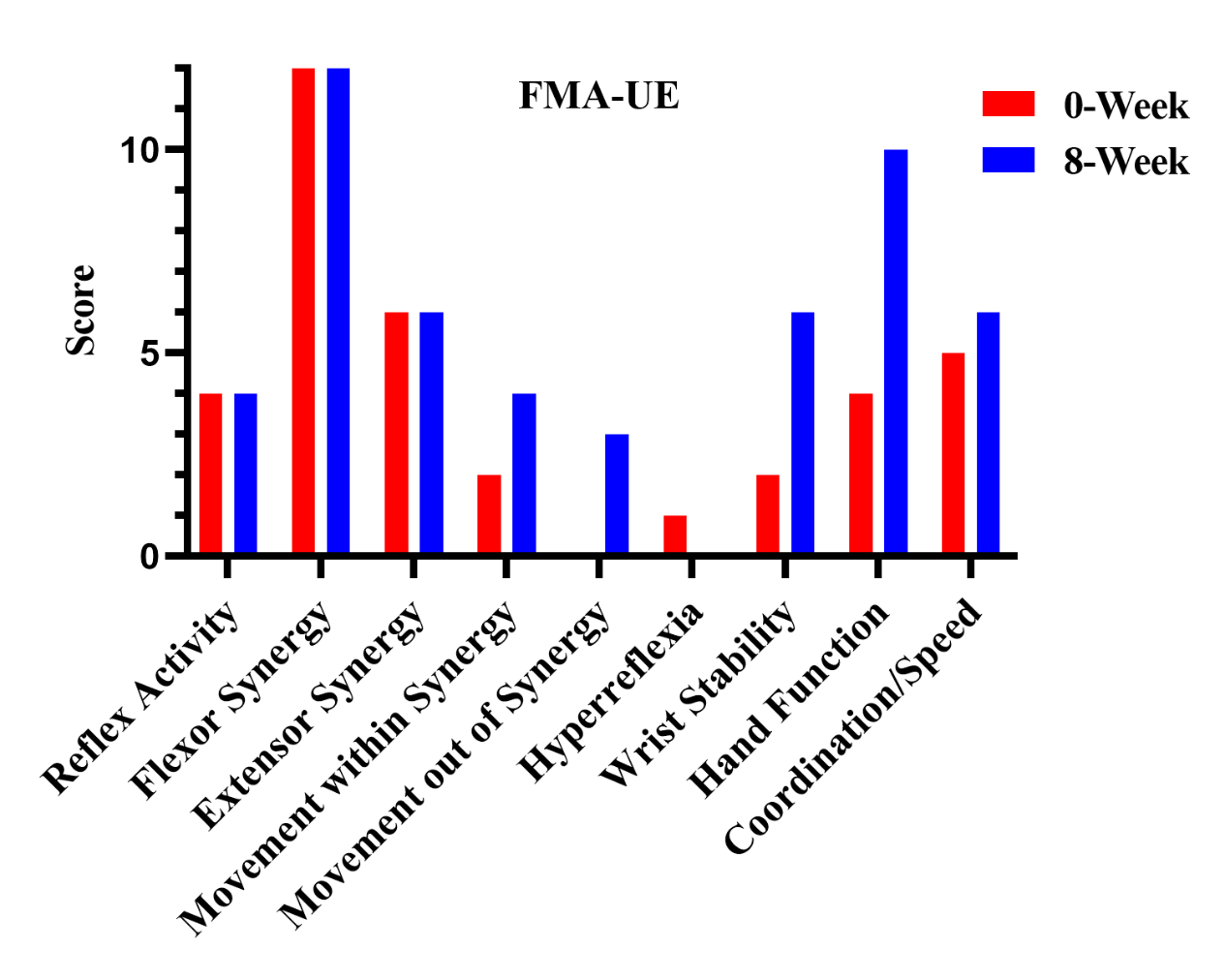
**

**Figure S1:** The Fugl-Meyer Assessment of Upper Extremity scores at 0-Week and 8-Week. FMA-UE, Fugl-Meyer Assessment of Upper Extremity.

**
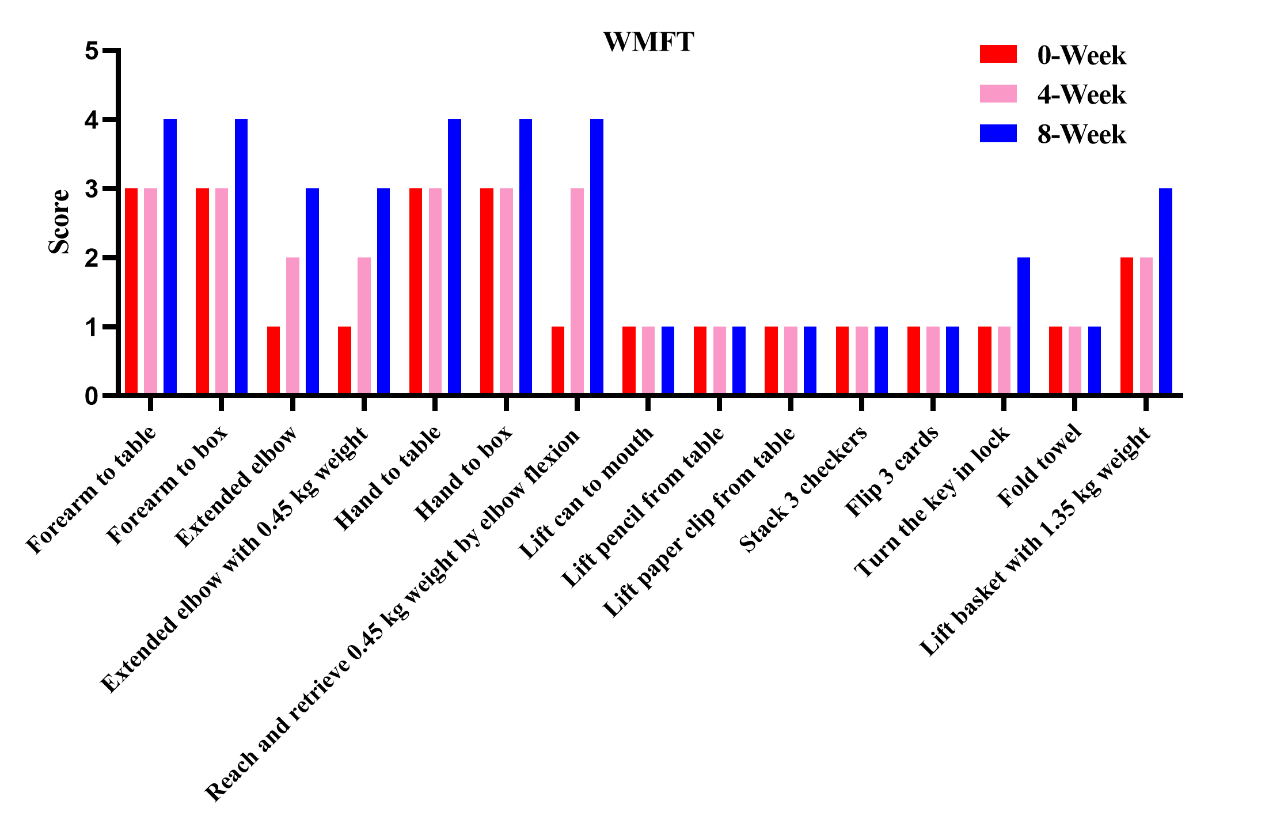
**

**Figure S2:** The Wolf Motor Function Test scores at 0-Week, 4-Week, and 8-Week (excluding strength tests). WMFT, Wolf Motor Function Test.

**References**

Hiragami, S., Inoue, Y., & Harada, K. (2019). Minimal clinically important difference for the Fugl-Meyer assessment of the upper extremity in convalescent stroke patients with moderate to severe hemiparesis. *J Phys Ther Sci*, 31(11), 917-921. doi:10.1589/jpts.31.917.

Lin, K. C., Hsieh, Y. W., Wu, C. Y., Chen, C. L., Jang, Y., & Liu, J. S. (2009). Minimal detectable change and clinically important difference of the Wolf Motor Function Test in stroke patients. *Neurorehabil Neural Repair*, 23(5), 429-34. doi:10.1177/1545968308331144.
