# Supplementary figures and images for: Function-specific repetitive transcranial magnetic stimulation for fine motor rehabilitation in chronic stroke: a case report
Source: Psychoradiology. 2025 Nov 18;6:kkaf033. doi: 10.1093/psyrad/kkaf033 (PMC12902691; doi:10.1093/psyrad/kkaf033)

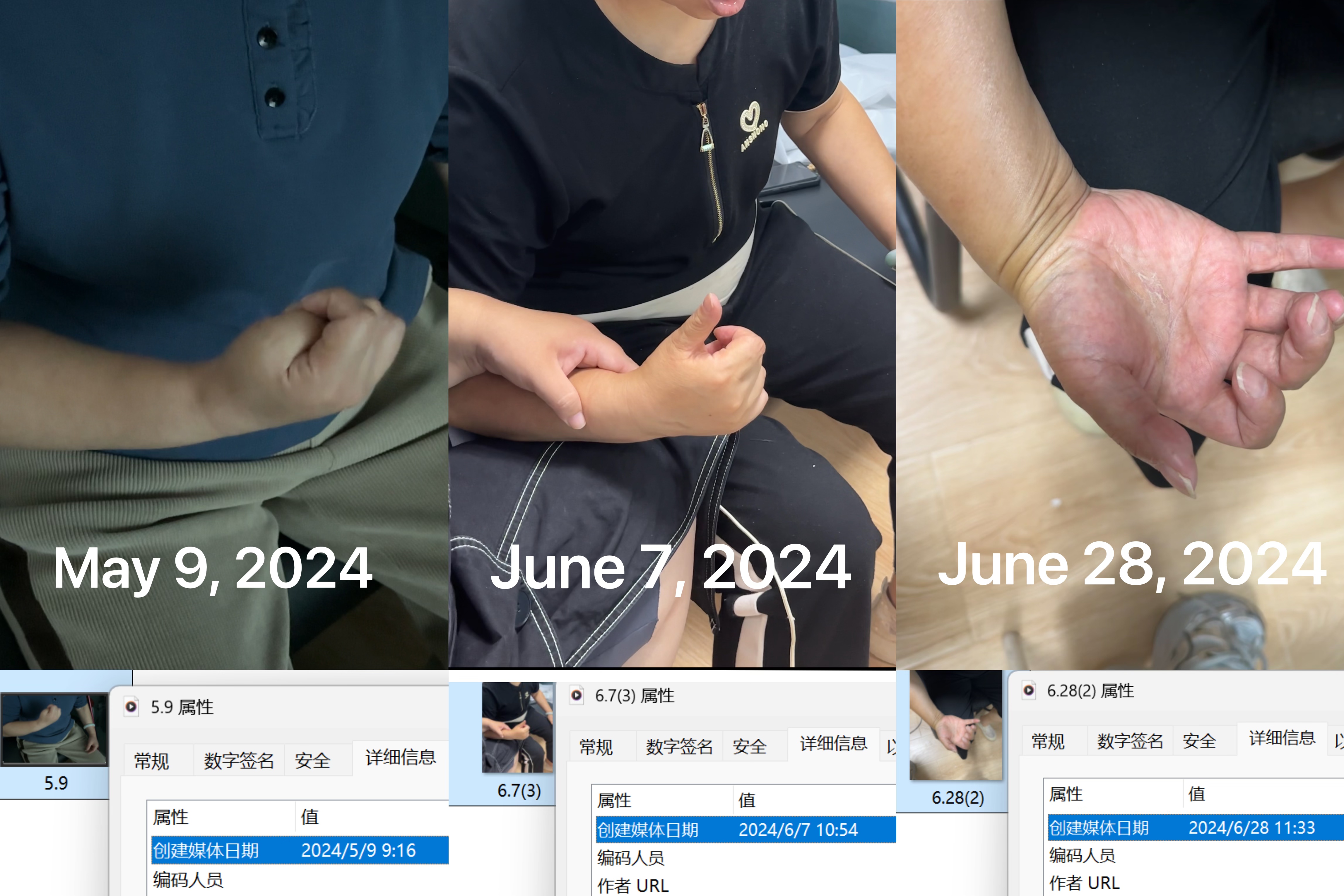

Supplement: kkaf033_Supplemental_Files [file kkaf033_supplemental_files.zip › Supplementary video thumbnail image.jpg]
